# Tumor Lipid Signatures Are Descriptive of Acquisition of Therapy Resistance in an Endocrine-Related Breast Cancer Mouse Model

**DOI:** 10.1021/acs.jproteome.3c00382

**Published:** 2023-07-27

**Authors:** Rita Araújo, Victoria Fabris, Caroline A. Lamb, Andrés Elía, Claudia Lanari, Luisa A. Helguero, Ana M. Gil

**Affiliations:** † Department of Chemistry and CICECO - Aveiro Institute of Materials (CICECO/UA), University of Aveiro, Campus Universitario de Santiago, 3810-193 Aveiro, Portugal; ‡ IByME − Instituto de Biología y Medicina Experimental, Vuelta de Obligado 2490, C1428 ADN Buenos Aires, Argentina; § iBIMED - Institute of Biomedicine, Department of Medical Sciences, Universidade de Aveiro, Agra do Crasto, 3810-193 Aveiro, Portugal

**Keywords:** breast cancer (BC), hormone receptor (HR) positive, metabolomics, nuclear magnetic resonance, lipids, membrane lipids, cholesterol, hormone-independent growth, endocrine therapy resistance

## Abstract

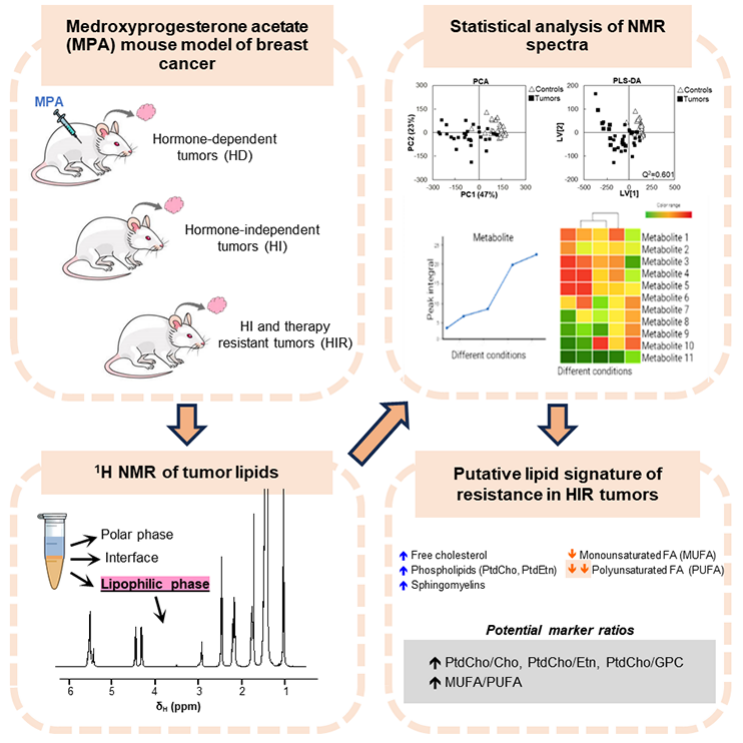

The lipid metabolism adaptations of estrogen and progesterone receptor-positive breast cancer tumors from a mouse syngeneic model are investigated in relation to differences across the transition from hormone-dependent (HD) to hormone-independent (HI) tumor growth and the acquisition of endocrine therapy (ET) resistance (HIR tumors). Results are articulated with reported polar metabolome results to complete a metabolic picture of the above transitions and suggest markers of tumor progression and aggressiveness. Untargeted nuclear magnetic resonance metabolomics was used to analyze tumor and mammary tissue lipid extracts. Tumor progression (HD-HI-HIR) was accompanied by increased nonesterified cholesterol forms and phospholipids (phosphatidylcholine, phosphatidylethanolamine, sphingomyelins, and plasmalogens) and decreased relative contents of triglycerides and fatty acids. Predominating fatty acids became shorter and more saturated on average. These results were consistent with gradually more activated cholesterol synthesis, β-oxidation, and phospholipid biosynthesis to sustain tumor growth, as well as an increase in cholesterol (possibly oxysterol) forms. Particular compound levels and ratios were identified as potential endocrine tumor HD-HI-HIR progression markers, supporting new hypotheses to explain acquired ET resistance.

## Introduction

Breast cancer (BC) is responsible for the highest number of cancer-related deaths in women, accounting for nearly 25% of diagnosed cancer cases in females. Most BC cases (ca. 70%) express estrogen receptor alpha (ERα) and/or progesterone receptor (PR), being designated as hormone receptor (HR) positive BC.^1^ HR-positive breast tumors depend on activated ERα to support their growth, and thus, most endocrine therapies (ET) target the ER signaling pathway,^2^ usually blocking ERα transcriptional activity with drugs such as tamoxifen or fulvestrant and using aromatase inhibitors.^3^ Tumor growth can also be sustained by PR activation, and some ETs have been developed to target this receptor.^4−6^ The most common progestins used for BC treatment, particularly at advanced stages, comprise megestrol acetate and medroxyprogesterone acetate (MPA),^6^ while antiprogestins (e.g., RU486 or mifepristone, and onapristone) have been used in clinical trials, but they are still not an option in the clinic.^7−9^ Moreover, recent findings from the MIPRA window of opportunity clinical trial support the use of mifepristone in patients with luminal breast cancer with high PR isoform A/isoform B ratios.^7^ However, most BC patients initially respond to ET but eventually relapse (acquired resistance) and about 20–30% do not respond (*de novo* resistance).^4^ Resistance to ET is accompanied by a range of cellular changes, for instance, overexpression of the MYC transcription factor,^4,10^ that are associated with metabolic adaptations. Hence, characterizing the metabolic changes associated with ET resistance may not only help to further understand the mechanisms of ET resistance but also unveil markers with potential predictive ability, which could assist the clinician in implementing improved personalized treatment schemes.

The syngeneic medroxyprogesterone (MPA)-induced BC mouse model^11^ has been extensively used as a model of HR-positive BC. This model is one of the few murine models that more closely resemble many aspects of the presentation and progression of HR-positive BC in humans,^11^ and hence, the MPA model is particularly useful to study the processes through which hormone-dependent tumors (HD tumors, grown in animals supplemented with an MPA depot) become independent of hormones for growth (HI tumors; still responsive to ET), and which eventually acquire ET resistance (HIR tumors). At the metabolic level, the above three types of tumors are characterized by distinct polar metabolomes^12^ indicative of different metabolic signatures accompanying each transition. For instance, the relative levels of glutamate, uridine diphosphate *N*-acetylglucosamine (UDP-GlcNAc), glycerophosphocholine (GPC), and lactate could differentiate the three tumor types, with HIR tumors exhibiting a stronger glycolytic profile, distinct *O*-glycosylation potential, and membrane metabolism characteristics, compared to HD and HI tumors. This was the first study characterizing the metabolic changes in the transition of HD tumors toward acquired ET resistance in a mouse model of HR-positive BC. As far as we know, no studies have been published regarding the lipidic composition of these tumors, and given that lipids are known as long-standing important players in cancer biology,^13,14^ such characterization is certainly an important avenue to pursue.

Indeed, numerous studies have established dynamic lipid phenotypes accompanying malignant transformation and tumor progression in different types of cancer, including breast cancer.^15−18^ Lipid metabolism is known to affect tumor growth at different levels, namely, through biochemical routes for energy regulation, and signaling mediators regulating invasion, metastasis, cell proliferation, and apoptosis.^13,14,19−21^ In the case of breast cancer, a number of highly expressed genes have been found to regulate lipid metabolism, for instance, unveiling phospholipids (PLs) as important players in breast cancer diagnosis and therapeutic protocols.^18^ Indeed, a range of studies performed either in BC cells, human biofluids, animal tissues, or human tissues, using techniques such as immunohistochemistry in tandem with lipidomic/metabolomic strategies based on mass spectrometry (MS) or nuclear magnetic resonance (NMR) spectroscopy, have demonstrated the relevance of specific lipids in distinguishing healthy subjects from BC patients,^22−36^ the relation to prognosis in different BC subtypes,^18,24^ or implications in treatment efficacy.^20,37−40^

Lipids have also been related to drug resistance in breast cancer cell lines and tumors.^41−44^ In a comparative study of sensitive and resistant MCF-7 cells, the latter showed an increased content of free cholesterol packed into enlarged lysosomes and of triglycerides (TGs) stored in large lipid droplets.^42^ In addition, when comparing those same cell lines in contact with differentiated 3T3-L1 adipocytes, to mimic the BC microenvironment, the levels of phosphatidylcholine (PtdCho), phosphatidylethanolamine (PtdEtn), ceramides (Cer), and sphingomyelins (SMs) were found to be relatively elevated in resistant cells.^43^ Furthermore, in an *in vitro* study comparing two ET-resistant BC cell lines (SUM 44PE LTED and MM134), the increased expression of SREBP, an activator of fatty acid (FA) and cholesterol synthesis, was identified as a promoter of resistance.^44^

To date, most lipidomic and metabolomic studies comparing ET-sensitive versus resistant phenotypes have been carried out in cell lines *in vitro*, and to our knowledge, no studies have been reported on progestin-dependent tumors. Therefore, this work aimed to characterize the lipid signatures associated with HR-positive mammary tumors, as tumors progress from HD to HI growth and, subsequently, acquire resistance to antiprogestins (HIR). Although MS has been most often associated with lipid metabolomics, we employ NMR spectroscopy metabolomics, exploiting its holistic nature and related ability to follow different lipid families simultaneously.

## Experimental Section

### Animal Model and Animal Experimentation

**Figure 1:**
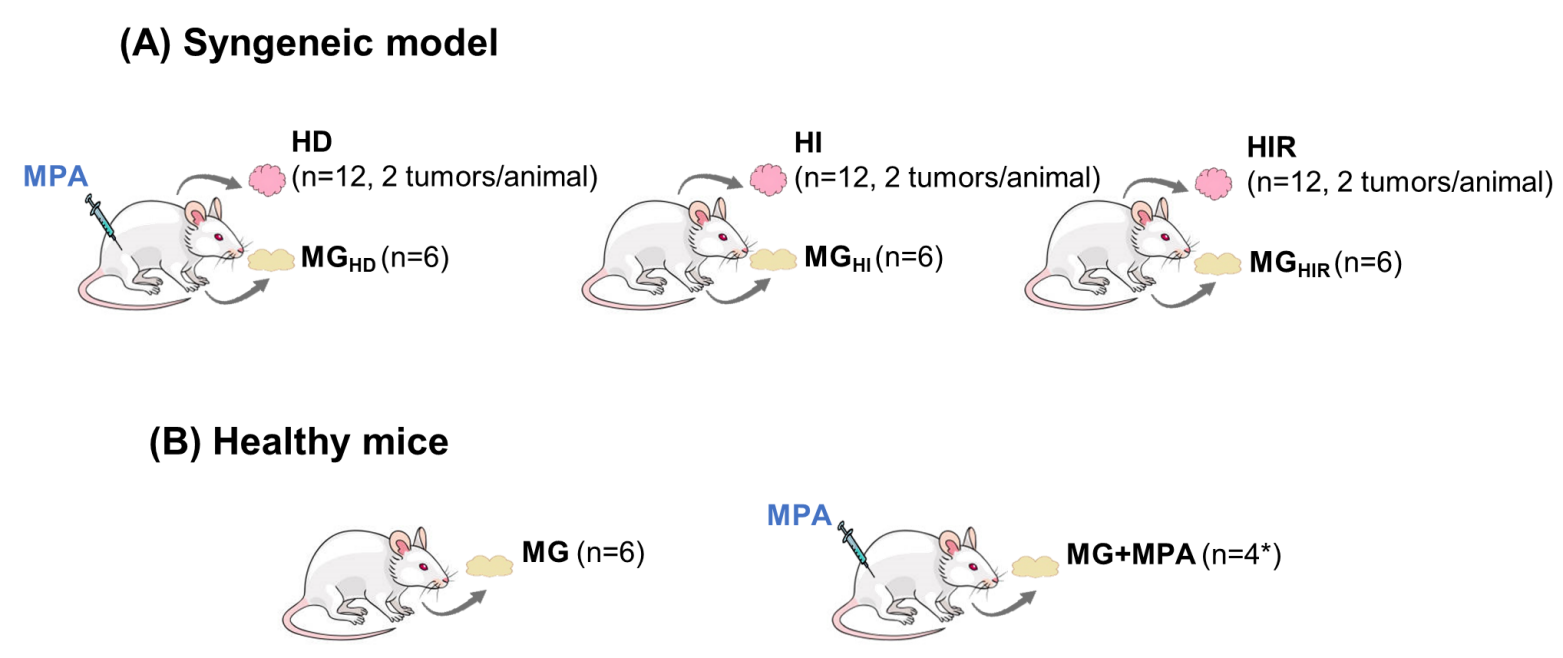
Experimental model and animal groups used in this study. (A) The mice transplanted with the syngeneic tumor lines were divided into groups of 6 mice, each implanted in the right and left inguinal flanks (*n* = 12 tumors from each tumor line); the HD group was implanted with the C4-HD line plus a 20 mg MPA depot, the HI group was implanted with the C4-HI line, and the HIR group was implanted with the C4-HIR line. When the tumor size reached 30–40 mm^2^ (*ca.* 16 days), tumor tissue (referred to as HD, HI, and HIR) and axillary mammary glands (MG, MG+MPA, MG_HD_, MG_HI_, and MG_HIR_) were excised for analysis. (B) The control animals (*n* = 12 mice) were assigned into two groups of 6 mice, consisting of no treatment or 20 mg of MPA depot *s*.*c*. (MG and MG+MPA, respectively); the mice were sacrificed after 16-day implantation, and their mammary glands were excised for analysis (* two of the MG+MPA samples could not be analyzed for technical reasons).

This work used the syngeneic mouse mammary ductal ER/PR-positive adenocarcinoma C4-HD and the derived tumor lines C4-HI and C4-HIR obtained through selective pressure by serial transplantation in mice without a MPA depot and with mifepristone treatment, respectively, as described elsewhere.^11^ Both C4-HD and C4-HI tumors regress if PR activation is blocked (either by MPA withdrawal (C4-HD) or by PR inhibition with antiprogestins (C4-HD and C4-HI). The C4-HIR tumor line grows in the presence of the antiprogestins mifepristone and onapristone.^11^ Two-month-old virgin female BALB/C mice were implanted subcutaneously with the C4-HD, C4-HI, and C4-HIR tumor lines into the right and left inguinal flanks of each mouse (*n* = 6 mice for each group; *n* = 12 tumors per group) (Figure 1A); these gave rise to tumors hereafter represented as HD, HI, and HIR, respectively. Tumors were allowed to grow to 30–40 mm^2^ (*ca.* 16 days) to obtain tumors growing in the exponential phase. Tumors and axial mammary gland (MG) tissue from the same mice were then excised and immediately frozen in liquid nitrogen. A matched cohort of mice without tumors was divided into two groups, one injected with 20 mg of MPA depot (healthy controls, designated as MG+MPA, *n* = 4, as two samples could not be analyzed for technical reasons), and the other left untreated (MG, *n* = 6) (Figure 1B).

The MG tissue was excised from these mice at the same time as the tumors from tumor-bearing mice, i.e., after ca. 16 days. All animals were maintained under a 12 h light/dark cycle and fed *ad libitum*. All animal experiments were approved by the local Institutional Animal Care and Use Committee (Approval No. 030/2016, dated June 24, 2016) and carried out in compliance with the regulatory standards of animal ethics. All animal procedures were performed at the Animal Facility at the Instituto de Biología y Medicina Experimental (IByME) of Buenos Aires, in Argentina.

### Sample Preparation and NMR Spectroscopy

Sample preparation was performed, ensuring that no sample thawing occurred during handling, before immediate methanol/water/chloroform extraction, as described previously.^11^ The lipophilic extracts were dried under nitrogen gas flow and stored at −80 °C. Before NMR analysis, the extracts were suspended in 600 μL of deuterated CDCl_3_ (99.8% deuterium), containing 0.03% tetramethylsilane (TMS) for chemical shift referencing. The samples were vortexed, and 550 μL was transferred to 5 mm NMR tubes. The unidimensional (1D) ^1^H NMR spectra of lipophilic extracts were acquired on a Bruker Avance III HD 500 spectrometer (Rheinstetten, Germany) operating at a frequency of 500.13 MHz for proton, at 298 K, using the *“zg”* pulse sequence (Bruker library), with 2.34 s acquisition time, 2 s relaxation delay, 512 scans, 7002.801 Hz spectral width, and 32 k data points. Each free-induction decay was zero-filled to 64 k points and multiplied by a 0.3 Hz exponential line-broadening function prior to Fourier transform. The spectra were manually phased, baseline-corrected, and chemical-shift-referenced to TMS (TopSpin software, 3.2 version, Bruker). Peak assignment was based on comparison with data available on the human metabolome database (HMDB),^45^ existing literature,^46−53^ 2D NMR experiments, and spiking experiments.

### Statistical Analysis

The 1D ^1^H NMR spectra were used to create a data matrix (AMIX-viewer 3.9.14, Bruker BioSpin, Rheinstetten, Germany) prior to multivariate analysis (MVA). The residual water (δ 1.46–1.69), methanol (δ 3.40–3.57), and chloroform (δ 7.02–7.51) signals were excluded from the matrix. Spectra were aligned by the recursive segment-wise peak alignment (Matlab 8.3.0, The MathWorks Inc.), normalized to spectral total area (Matlab 8.3.0, The MathWorks Inc.), and scaled by unit variance (UV) (SIMCA-P 11.5, Umetrics, Umeå, Sweden). MVA was carried out using principal component analysis (PCA) and partial least-squares discriminant analysis (PLS-DA) (SIMCA-P 11.5, Umetrics, Umeå, Sweden). Results were visualized through factorial coordinates (“scores plots”) and factorial contributions (“loadings plots”), the latter colored according to variable importance to the projection (VIP, Matlab 8.3.0, The MathWorks Inc.). All PLS-DA models were characterized by predictive power (or *Q*^2^) values, robust models corresponding to *Q*^2^ ≥ 0.5. Relevant resonances were integrated (Amix3.9.5, Bruker BioSpin, Rheinstetten, Germany), normalized to total spectral area, and assessed for statistical significance (*p*-values calculated by the Student’s *t* test, R-statistical software, R Foundation for Statistical Computing, Vienna, Austria). Significant changes in metabolite levels (*p* < 0.05) were identified and *p*-values corrected for false discovery rate (FDR) for multiple comparisons using the Benjamini and Hochberg method.^52^

## Results

### ^1^H NMR Spectra of Tumor Lipid Extracts

**Figure 2:**
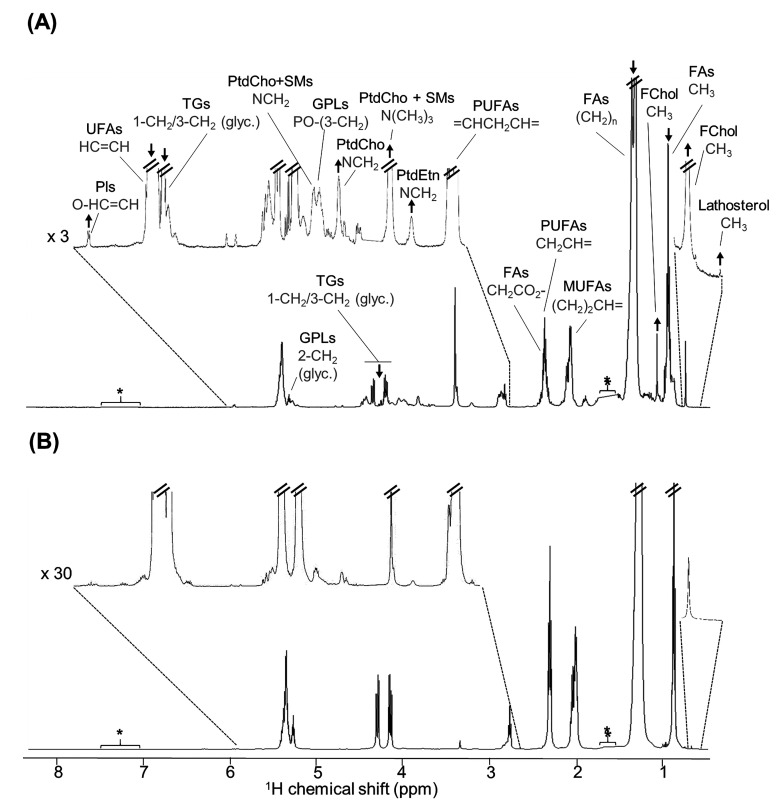
Normalized representative ^1^H NMR (500 MHz) spectra of lipid extracts of (A) an HD (+ MPA) tumor sample and (B) a MG sample (from a healthy mouse). Peak assignments are all shown in the top spectrum, distributed by the full spectrum and the insets, for the sake of clarity. Abbreviations: FAs, fatty acids; FChol, free cholesterol; glyc., glycerol; GPLs, glycerophospholipids; MUFAs, monounsaturated fatty acids; Pls, plasmalogens; PtdCho, phosphatidylcholine; PtdEtn, phosphatidylethanolamine; PUFAs, polyunsaturated fatty acids; SMs, sphingomyelins; TGs, triacylglycerides; UFAs, unsaturated fatty acids. Arrows identify visual peak variations between tumor and mammary gland samples. *, exclusion of spectral regions δ 1.69–1.46 and δ 7.47–7.02 corresponding to residual water and chloroform, respectively.

As expected, a representative ^1^H NMR spectrum of the lipid extracts of an HD tumor (Figure 2A) can be clearly distinguished from that of MG tissue obtained from a healthy animal (Figure 2B and Table S1). It is clear that the HD tumor tissue (as also observed for HIR and HIR tumors, spectra not shown) is richer in free cholesterol (FChol) and phospholipids (PLs) (namely, PtdCho, SMs, PtdEtn, and plasmalogens (Pls)) and poorer in TGs and FAs, compared to MG tissue, which is due to the high proportion of adipocytes in the latter. In the tumor, FChol predominates over esterified forms (not detected), and its precursor lathosterol is detected in low levels only in tumor samples (high field inset in Figure 2A, Table S1). It should be noted that the cholesterol peak at δ 1.00 corresponds to the 19-CH_3_ protons, which are not expected to be sensitive to hydroxylation in the 17C aliphatic chain, therefore potentially arising from both original and/or oxysterol forms of cholesterol as these oxidized forms hold great relevance in the context of BC (as will be discussed below). Also, in the tumor, PtdCho is the most abundant phospholipid, followed by SMs, PtdEtn, and Pls. The profile of unsaturated FA resonances (e.g., at δ 2–3 and ca. δ 5.3) (Figure 2) reflects a change in the FAs unsaturation pattern. In terms of specific FAs, only linoleic acid (LA) was detected, contrary to similar studies of different BC tumors, which reported the presence of other specific FAs (namely, oleic, linolenic, arachidonic, and docosahexaenoic).^28,53^

### Tumor Lipid Profiles Throughout HD-HI-HIR Progression

**Figure 3:**
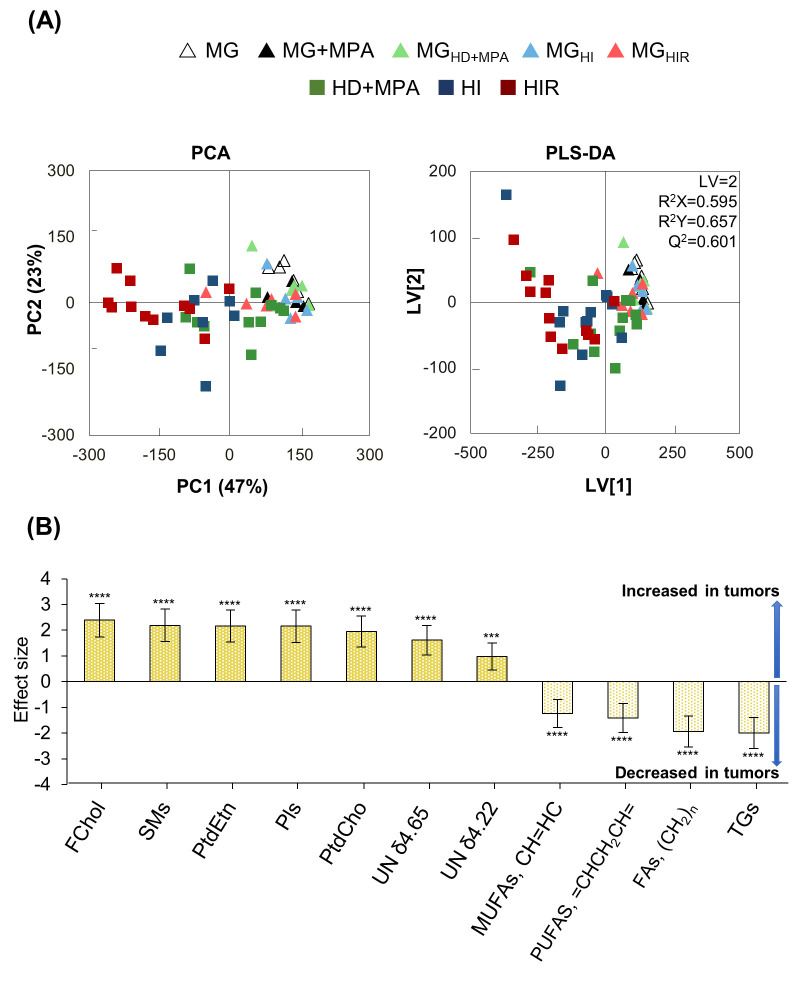
PCA and PLS-DA of spectra obtained for tumor samples compared to mammary gland samples, and corresponding overall metabolite differences. (A) PCA (left) and PLS-DA (right) scatter plots of all tumor samples (squares, total of *n* = 36) (HD+MPA, green, *n* = 12; HI, blue, *n* = 12; HIR, dark red, *n* = 12) versus all MG samples (triangles, total of *n* = 28) (MG, open, *n* = 4; MG+MPA, filled black, *n* = 6; MG_HD+MPA_, light green, *n* = 6; MG_HI_, light blue, *n* = 6; MG_HIR_, red, *n* = 6). (B) Main metabolite variations expressed by effect size. Abbreviations: FChol, free cholesterol; MUFAs, monounsaturated fatty acids; Pls, plasmalogens; PtdCho, phosphatidylcholine; PtdEtn, phosphatidylethanolamine; PUFAs, polyunsaturated fatty acids; SFAs, saturated fatty acids; SMs, sphingomyelins; TGs, triacylglycerides; UFAs, unsaturated fatty acids; UN, unassigned resonance. *, *p*-value <0.05; **, *p*-value <0.01; ***, *p*-value <0.001; ****, *p*-value <0.0001.

In order to assess if MPA injection may be a confounder by altering lipid profiles, MG versus MG+MPA comparison was first considered (Figure S1), having revealed no group separation in PCA or PLS-DA (*Q*^2^ < 0), although peak integration unveiled decreased levels of FChol (effect size −1.71 ± 1.47, *p*-value 0.019) in MG+MPA. Similarly, the presence of tumors in the inguinal flanks did not significantly influence the lipid profile of the axiliary MG (PLS-DA of [MG/MG+MPA] versus [MG_HD_/MG_HI_/MG_HIR_] with *Q*^2^ < 0), except for a weak increase in FChol noted in the MG of tumor-bearing animals (0.82 ± 0.81, *p*-value 0.018), with a particular enhancement for MG_HIR_ samples. A PCA scores plot of the ^1^H NMR spectra of all samples (Figure 3A, left) shows that all MG samples (triangles) are almost exclusively overlapped in positive PCA (except for some slightly more dispersed MG_HIR_ samples), while the lipid profiles of HD, HI, and HIR tumors (squares) follow a trajectory toward gradually more negative PC1. As expected, PLS-DA (Figure 3A, right) produced a robust model separating tumors from MG tissues (*Q*^2^ = 0.601), which confirmed that irrespective of tumor type, tumors are richer in FChol, PtdCho, SMs, PtdEtn, and Pls (and unassigned resonances at δ 4.65 and 4.22), while showing depletion in MUFAs, PUFAs, and TGs (Figure 3B), compared to the lipid profile of whole MG tissue.

**Figure 4:**
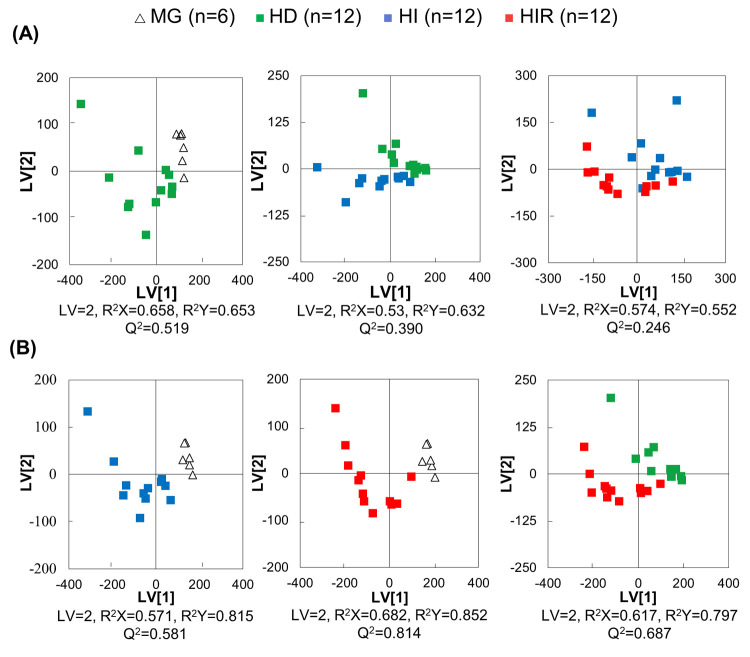
PLS-DA scatter plots for pairwise sequential and nonsequential group comparisons. (A) PLS-DA for sequential group comparisons: HD tumors versus MG tissue (healthy animals), HI tumors versus HD tumors, and HIR tumors versus HI tumors. (B) PLS-DA of nonsequential group comparisons: HI tumors versus MG tissue, HIR tumors versus MG tissue, and HIR tumors versus HD tumors. Legend: MG, open triangles (*n* = 6); HD, green squares (*n* = 12); HI, blue squares (*n* = 12); HIR, red squares (*n* = 12).

More importantly, pairwise PCA (Figure S2A) and PLS-DA (Figure 4A) analysis showed significant group separation between controls and HD tumors (PLS-DA *Q*^2^ = 0.519), with weaker models obtained for HD/HI/HIR tumors comparisons (PLS-DA *Q*^2^ = 0.390 and 0.246). HD tumors are distinguished from MG tissue by significantly increased FChol (thus compensating any cholesterol-lowering effects of MPA) and PLs, and decreased FAs (specifically including PUFAs) and TGs (Table 1, top section). HI tumors are distinguished from HD tumors solely by further increased levels of PtdEtn, SMs, and Pls (and PtdCho levels not significantly changed). Compared to HI tumors, HIR tumors show additional increases in PtdCho, SMs, and Pls (and not significantly changed PtdEtn levels), in tandem with increased FChol and decreased FAs, as viewed by the global (CH_2_)_*n*_ resonance (Table 1, top section).

**Table 1:**
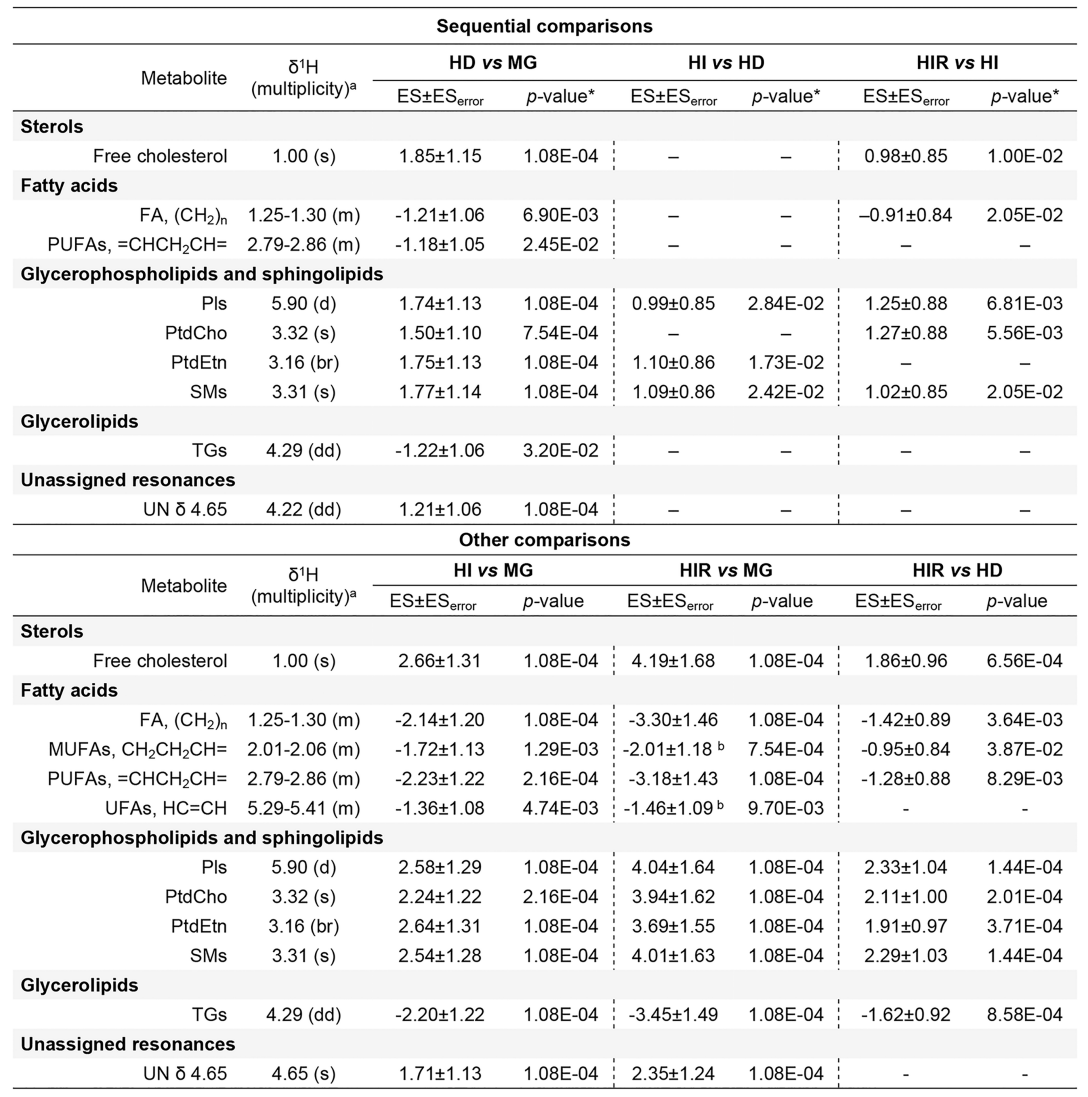
Statistically Significant Lipid Changes Found in Pairwise Sequential (Top Section) and Nonsequential (Bottom Section) Group Comparisons: MG Tissue (Healthy Animals) to HD (+MPA) Tumors, HD to HI Tumors, and HI to HIR Tumors^a^

a Abbreviations: effect size (ES); FAs, fatty acids; MUFAs, monounsaturated fatty acids; Pls, plasmalogens, PtdCho, phosphatidylcholine; PtdEtn, phosphatidylethanolamine; PUFAs, polyunsaturated fatty acids; SMs, sphingomyelins; TGs, triacylglyceride; UFAs, unsaturated fatty acids. multiplicity: s, singlet; d, doublet; dd, doublet of doublets; ddd, doublet of doublets of doublets; t, triplet; q, quartet; m, multiplet; br, broad signal. All variations shown remained significant after FDR correction. U_δ_: unassigned signal at chemical shift δ. Superscript “a” in table body indicates the peak used for integration (part of the spin system). *, All *p*-values shown were obtained after FDR correction.

Nonsequential comparisons give rise to more robust models (Figures S1B and 4B, for PCA and PLS-DA Q^2^ 0.58–0.81, respectively), indicating that MG tissue is clearly differentiated from both HI and HIR tumors (Table 1, bottom section), with basis on more enhanced changes in FChol and PLs (increases) and in FAs and TGs (decreases), revealing MUFAs and general UFAs resonances (not identified before in sequential analysis) as part of the distinguishing signatures. HIR tumors are clearly differentiated from HD tumors (PLS-DA with Q^2^ > 0.5) (Figure 4B) through increased FChol and PLs and decreased FAs and TGs (Table 1, bottom section).

**Figure 5:**
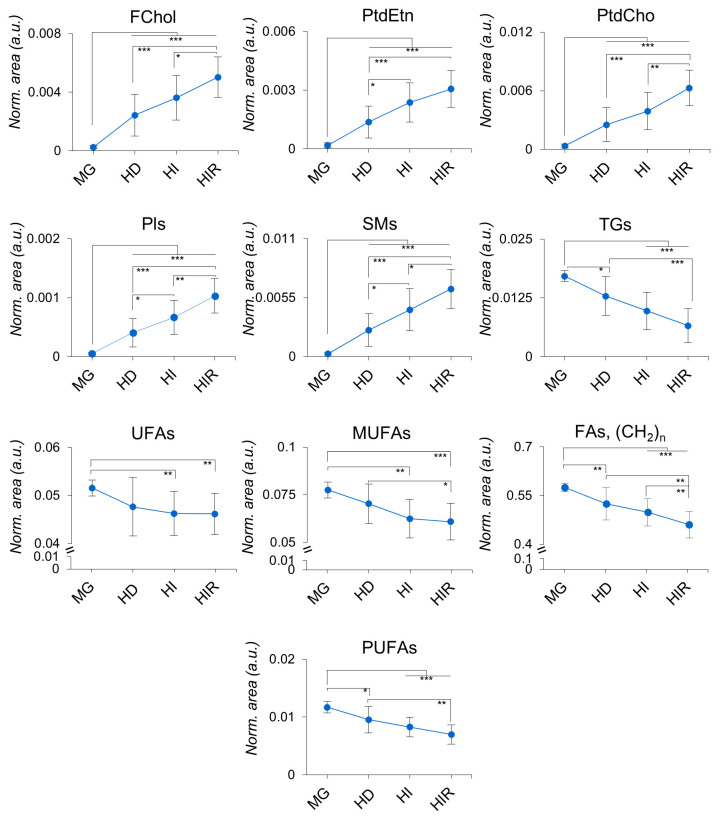
Lipid resonances trajectories throughout tumor stage progression. Peak areas corresponding to each lipid moiety were normalized by total spectral area. MG, mammary gland from healthy mice; HD, hormone-dependent tumor; HI, hormone-independent tumor; HIR, hormone-independent and therapy-resistant tumor. Abbreviations: FChol, free cholesterol, FAs, fatty acids; MUFAs, monounsaturated fatty acids; Pls, plasmalogens; PtdCho, phosphatidylcholine; PtdEtn, phosphatidylethanolamine; PUFAs, polyunsaturated fatty acids; SMs, sphingomyelins; TGs, triacylglycerides; UFAs, unsaturated fatty acids. *, *p*-value <0.05; **, *p*-value <0.01; ***, *p*-value <0.001; ****, *p*-value <0.0001.

**Figure 6:**
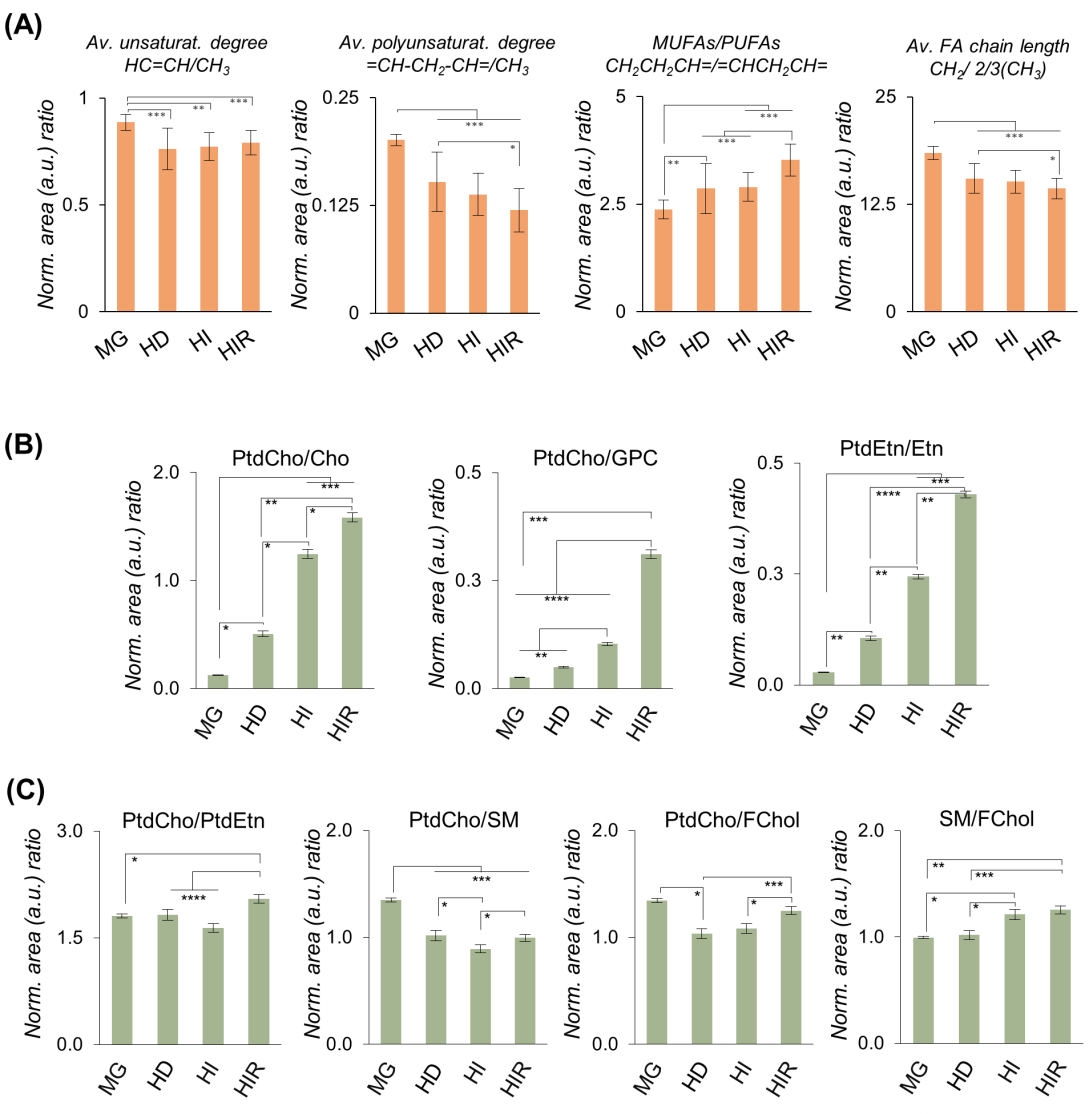
Average FA characteristics and selected lipid ratios. (A) Average FA unsaturation degree, average FA polyunsaturation degree, MUFAs/PUFAs ratio, and average FA chain length. (B) Selected area ratios of phospholipids with respective precursors (the latter areas were obtained from the spectra of polar extracts of the same samples^12^ so that only ratio evolution should be considered, rather than absolute ratio values): PtdCho/Cho, PtdCho/GPC, PtdEtn/Etn. (C) Selected area ratios for membrane lipids: PtdCho/PtdEtn, PtdCho/SM, PtdCho/FChol, and SM/FChol. Abbreviations as defined in Figure 1 caption. *, *p*-value <0.05; **, *p*-value <0.01; ***, *p*-value <0.001; ****, *p*-value <0.0001.

Hence, the levels of lipid families identified here serve as clear distinctive signatures of all tumor groups under study, as better illustrated in trajectory plots (Figure 5). It should be noted that lipids were not quantified in absolute, as spectra were not recorded in quantitative conditions, only relative quantities or ratios being discussed below. The trajectories of normalized areas (Figure 5) clearly indicate the relative lowering of MUFAs and PUFAs from MG to tumors, along with the general resonance arising from all UFAs. This decrease is expected as MG tissue is richer in adipocytes containing higher amounts of TGs, and hence not deemed biologically relevant. Still, it is interesting to note the decreasing average of unsaturation degree in tumors (Figure 6A), with PUFAs showing a decreasing tendency across tumor progression (HD-HI-HIR), capable of differentiating HD/HIR tumors. Interestingly, the MUFAs/PUFAs ratio follows the MG < HD = HI < HIR trend, which expresses a stronger depletion in PUFAS, compared to that in MUFAs (Figure 6A and Table S2). These changes are accompanied by a decrease in average chain length following an inverse trend: MG > HD = HI > HIR.

Hence, progression seems accompanied by increased levels of shorter and more saturated FAs. Regarding membrane phospholipids (Figure 6B), and making use of their polar precursor contents as measured in the aqueous extracts of the same samples,^12^ it becomes clear that PtdCho increases throughout progression, at the expense of Cho and mainly GPC (lower PtdCho/GPC ratio range, compared to PtdCho/Cho) (Figure 6B and Table S2), with a similar behavior observed for PtdEtn and Etn. (Note that the graphs in Figure 6B should only be read in terms of ratio variations, rather than absolute ratio values since resonances from different spectra, polar and lipidic extracts, are compared.)

However, the proportion of PtdCho to PtdEtn (as expressed by PtdCho/PtdEtn, and with a clear abundance of PtdCho) is maintained constant except for an increase from HI to HIR tumors (Figure 6C and Table S2). The content of SMs relative to PtdCho (or PtdEtn, not shown), as viewed by the inverse PtdCho/SM ratio (Figure 6C), increases significantly from MG to HD, and then slightly to HI tumors, going back to HD values in HIR tumors. Pls were not used for ratio calculation due to the low signal-to-noise. As FChol incorporates cell membranes (although not exclusively), the evolution in PtdCho/FChol and SM/FChol ratios may be indicative of membranes becoming proportionally richer in PLs, relative to FChol, across tumor progression.

## Discussion

### Fatty Acid Metabolism

**Figure 7:**
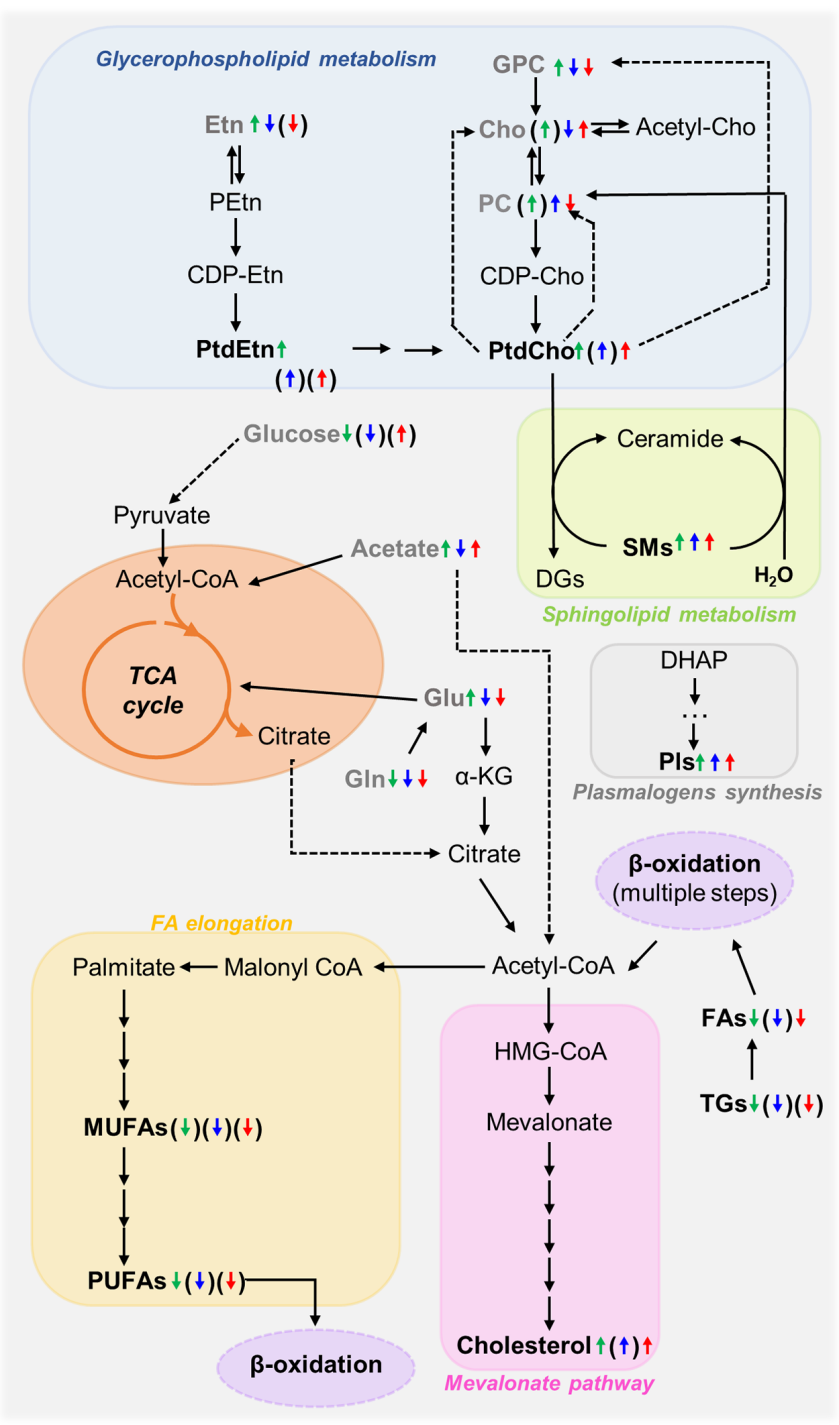
Putative metabolic pathways identified as the main metabolic adaptations of lipid contents across MG-HD-HI-HIR tissues. Compound names shown in bold identify compounds detected by NMR in this work and as reported previously,^12^ irrespective of their variation. Gray-colored metabolites identify polar metabolites identified to vary in the polar extracts of the same samples considered here.^12^ The colored arrows (↓↑) illustrate significant or qualitative tendencies (with no statistical significance, in brackets) found in each pairwise comparison: HD versus MG (green left arrow), HI versus HD (blue middle arrow), and HIR versus HI (red right arrow). Abbreviations: α-KG, alpha-ketoglutarate; CDP-Cho, cytidine diphosphate choline; CDP-Etn, cytidine diphosphate ethanolamine; Cho, choline; DG, diacylglycerides; DHAP, dihydroxyacetone phosphate; Etn, ethanolamine; FAs, fatty acids; MUFAs, monounsaturated FAs; Gln, glutamine; Glu, glutamate; GPC, glycerophosphocholine; HMG-CoA, 3-hydroxy-3-methylglutaryl coenzyme A; PC; phosphocholine; PEtn, phosphoethanolamine; Pls, plasmalogens; PtdCho, phosphatidylcholine; PtdEtn, phosphatidylethanolamine; PUFAs, polyunsaturated FAs; SMs, sphingomyelins; TGs, triacylglycerides.

Changes in FA pathways were expressed in their average chain length and saturation degree, both between MG and tumor tissue and, more importantly, between HD, HI, and HIR phenotypes. Tumor HD-HI-HIR progression was characterized by increased levels of shorter chain FAs and a decrease in unsaturated FAs (particularly PUFAs). This is consistent with enhanced β-oxidation targeting PUFASs more significantly and leading to their faster decline, compared to the slower declining MUFA (Figure 7). This hypothesis is supported by a previous microarray analysis comparing the C4-HI to C4-HD tumors analyzed here.^54^ Indeed, in HI tumors there was an upregulation of peroxisomal biogenesis (PEX3, PXMP2) and β-oxidation enzymes PECR, ACOX1, and ACSL3 acting in the peroxisome and the mitochondrial ACADS. It is interesting, however, that the levels of the reduced form of glutathione (GSH) did not vary consistently across HD-HI-HIR progression, peaking in HI tumors and decreasing back to HD levels, in HIR tumors, as shown by polar extract analysis.^12^ This suggests that oxidation mechanisms in HD-HI-HIR progression may not necessarily be accompanied by a consistent engagement of glutathione-related pathways, which may thus not be robust distinguishers of HIR tumors, relative to both HD and HI tumors. We postulate, therefore, that the lower PUFAs content in HIR, compared to HD and HIR tumors, does evidence increased β-oxidation, although the corresponding mechanisms (and relation to GSH levels) require further understanding.

We hypothesize that acetyl-CoA resulting from lipid oxidation will serve two outcomes (Figure 7): to enter the TCA cycle for enhanced energy production (since we previously showed that HI and HIR tumors produce higher lactate than HD tumors and rely on glutaminolysis and anaplerotic amino acids feeding to support the TCA,^12^ and to enter cholesterol biosynthesis through the mevalonate pathway to support the demand for cholesterol accumulation either at the membrane or as a metabolic precursor of oxysterols as discussed below. We further suggest that it is possible that the predominating shorter FAs in tumors (particularly in HIR tumors) may be incorporated into the newly synthesized membrane PLs, thus reversing (at least in part) the fluidity decrease expected to arise from increasing cholesterol (discussed below). The observed decreases in the resonances from TGs and FA methylene protons across the HD to HI, and to HIR transitions seem to mainly reflect the effects of enhanced β-oxidation, using up storage lipids and decreasing FA chain length (eventually giving rise to acetyl-CoA) (Figure 7). Previous reports have shown upregulation of FA synthase (FASN) in breast cancer,^44^ including LTED cells. If confirmed in tumors, this would contribute to FA synthesis, probably to compensate for the higher β-oxidation demands. Interestingly, although FASN upregulation has been correlated to chemotherapy resistance, its connection with endocrine resistance remains unclear.^44^

### Cholesterol Metabolism

One of the main observations in this study regards the increase of FChol levels in all tumors, compared to MG, and particularly from HI to HIR tumors, with evidence that HIR tumors may already be stimulating cholesterol synthesis/uptake in their environment (higher cholesterol contents in MG_HIR_ samples). Cholesterol metabolism activation is consistent with the detection of its precursor lathosterol in the tumors, and not in MG samples. There is vast evidence that increased cholesterol levels accompany cancer development, relying on *de novo* biosynthesis from acetyl-CoA in the endoplasmatic reticulum.^55^ This involves the expression of many enzymes regulated by sterol regulatory element binding protein (SREBP) transcription factors, with a direct impact on intracellular cholesterol levels.^56^ In breast cancer, TP53-mediated cholesterol synthesis through the SREBP pathway has been related to cell proliferation increase and self-renewal, involving the prenylation of Rho GTPases.^57^ The significant increase in FChol observed from HD to HI, and from HI to HIR tumors, in the present study, is thus consistent with the activation of sterols metabolism in endocrine-resistant ER-positive cells and was supported by a previous microarray analysis comparing the C4-HI to C4-HD tumors analyzed here.^54^ Although this microarray analysis did not include HIR tumors, it revealed a higher expression of cholesterol biosynthetic pathways enzymes in HI tumors compared to that in HD,^54^ namely, 3-hydroxy-3-methylglutaryl-Coenzyme A synthase 1 (HMGCS1), which catalyzes the condensation of acetyl-CoA with acetoacetyl-CoA to form HMG-CoA, converted into the cholesterol precursor mevalonate (Figure 7). We propose that the gradual cholesterol increase during tumor progression may be occurring through the mevalonate pathway, supported by glutamine and acetate, to enhance acetyl-CoA levels^12,58^ (Figure 7). In HI tumors, the increase in cholesterol 25-hydroxylase (CH25H) involved in the biosynthesis of 7-alpha, 25-dihydroxycholesterol (7-alpha, 25-OHC) and insulin-induced gene 1 protein (Insig1)^59−61^ that block the activity of SREBPs further suggests feedback control of cholesterol synthesis.

Increased free cholesterol levels may serve several ends, from decreasing membrane fluidity to involvement in oncogenic signaling activation (through membrane receptors) and cellular signal transduction through lipid rafts. Furthermore, its role in cell proliferation has been demonstrated in long-term estrogen-deprived (LTED) cells (MM134 and SUM44 BC cell lines) and were reported to activate FA/cholesterol metabolisms with 25-hydroxycholesterol (25HC) and 27-hydroxycholesterol (27HC) driving cell proliferation;^44,62^ the latter compound has been confirmed to impact BC pathogenesis by the selection of cells resistant to ferroptosis.^63^ Cholesterol is also a precursor of bile acids (although these were not detected in this study) and steroid hormones in endocrine-related breast and prostate cancers.^64^ Indeed, HI tumors expressed higher levels of the bile acid metabolism enzymes 24-hydroxycholesterol 7-alpha-hydroxylase (Cyp39a1), ATP-binding cassette subfamily C member 3 (Abcc3), and 3-hydroxyacyl-CoA dehydrogenase type-2 (Hadh2, involved in steroid hormones and bile acid metabolisms). HI tumors also expressed higher low-density lipoprotein receptor adapter protein 1 (Ldlrap1) and apolipoprotein E (ApoE) required for efficient endocytosis of low-density lipoprotein receptor (LDLR); therefore, the higher proportion of FChol measured in HI versus HD tumors may also be explained by upregulation of these proteins. Taken together, our results support the hypothesis that when tumors become independent of progestins to grow, they remodel their metabolism to enhance cholesterol biosynthesis and the uptake of free (nonesterified) cholesterol forms (which may include 25HC and 27HC, for instance). In T47-D, MCF-7 cells, and C4-HI tumors, FGF2 induces ERα and PR interaction at the CCND1 and cMYC DNA regulatory elements to support proliferation.^65^ It remains to be established if 25HC and 27HC contribute to this interaction to promote HI growth, for instance, through liver-S-receptor (LXR) recruitment to the same sites, as it has previously been shown to interact with ERα.^4,66^ A similar analysis has not been carried out for HIR tumors; however, our results indicate higher FChol levels compared to HI tumors, possibly due to the enhancement of the mechanisms described above and, thus, becoming part of a potential specific signature of acquired resistance.

### Phospholipid Metabolism

The overall increase in PLs and PLs/precursor ratios from MG to HD and then throughout tumor HD-HI-HIR progression might reflect increased cell proliferation. However, as all tumors were excised in the exponential growth phase, comparable degrees of cell proliferation were expected and confirmed as equivalent for all tumors (Figure S3). Therefore, we hypothesize that PLs’ increases are needed to sustain tumor growth, both by enhancing membrane biosynthesis, including for endoplasmic reticulum capacity enhancement and particularly to support extended protein glycosylation.^12^ Hence, increased PL levels (namely, PtdCho, SMs, PtdEtn, and Pls, in decreasing order of abundance) are confirmed as differentiators of tumoral tissue from healthy tissue and of tumors of increasing aggressiveness, as reported in previous studies of BC.^18,67−69^ However, to our knowledge, these changes were here observed for the first time as accompanying the HD to HI, and to HIR, transitions in the MPA mouse model of BC. Indeed, in HIR tumors, these increases are consistent with the lowest GPC levels previously reported for the same tumors^12^ (Figure 7). PLs ratios show that PtdCho remains the most abundant PL, maintaining its proportion to PtdEtn, except for a slight increase for HIR tumors, making higher PtdCho/PtdEtn values an HIR distinguishing feature. SMs are also important components in the plasma membrane and other structures such as the endocytic recycling compartment and the Golgi network,^70^ and their small enrichment, compared to that of PtdCho, from MG to HD, may reflect cholesterol integration in membrane lipid rafts, stabilized by SMs.^70^ Throughout tumor progression, both SM and PtdCho increase in similar proportions, as shown by the almost unaltered PtdCho/SM ratio, which suggests that no significant changes take place in the composition of SM/cholesterol lipid rafts within the tumors (although HI tumors seem slightly enriched in SMs). The increases in these most abundant PLs are slightly more pronounced than that of cholesterol, as seen by increasing ratios of PtdCho/FChol and SM/FChol, which may potentially serve as markers of tumor aggressiveness from HD/HI to HIR and from HD to HI/HIR, respectively. Regarding plasmalogens increase (probably arising from dihydroxyacetone phosphate, DHAP, although the corresponding biosynthetic enzymes did not appear upregulated in the microarray analysis of HD and HI)^54^ (Figure 7), it has been reported that plasmalogen levels are higher in cancer cells compared to those in healthy cells and the possible reasons for this have been recently reviewed.^71^ The ether-linkage lipase-stable lipids are believed to play an important role in membrane organization, facilitating membrane diffusion processes and possibly contributing to lipid raft microdomains. Plasmalogens also display antioxidant properties, which is consistent with their higher levels in HIR tumors in which shorter FAs are found, as a result of enhanced β-oxidation.

Overall, the results above indicate that HI tumors seem to be effectively differentiated from HD and HIR tumors by (i) intermediate levels of SMs and Pls and (ii) increased levels of PLs (PtdCho or PtdEtn/precursors, in tandem with lower PtdCho/PtdEtn and PtdCho/SM ratios). In addition, acquisition of ET resistance, i.e., HIR tumors, may be clearly differentiated from the remaining tumors by (i) increased levels of FChol, PtdCho, SMs, and PLs, and decreased global FA levels; as well as by (ii) increased MUFAs/PUFAs and PLs (PtdCho or PtdEtn/precursors and PtdCho/PtdEtn ratios). Notably, higher PtdCho/SM ratios distinguish HIR from HI (but not from HD), and higher PtdCho/FChol distinguishes HIR from HD and HI (but not from MG). These observations require complementation by absolute quantitation of the relevant lipid species, to confirm and establish absolute levels of distinguishing markers for HI and HIR tumors.

## Conclusions

General observations characterizing lipid profile differences across tumor progression from HD to HI, and then to HIR tumors, comprised increased FChol and PLs (PtdCho, PtdEtn, SMs, and Pls) and decreases in the relative contents of TGs and FAs. In addition, in the tumors, FAs became shorter and more saturated on average, comprising decreasing amounts of MUFAS and, particularly, of PUFAs. Taken together, these results were consistent with activated cholesterol synthesis, β-oxidation, and PLs biosynthesis to sustain tumor growth, with process dynamics defining specific signatures and parameters capable of distinguishing within different tumor types, as to their need of hormone for growth (HD to HI) and their acquired resistance to endocrine therapy (HI to HIR). The HD to HI transition seemed mainly described by adaptations in phospholipid metabolism, mostly connected to membrane dynamics and composition, whereas the acquisition of endocrine therapy resistance (HI to HIR) was characterized by a distinctive lipid signature of further increased PLs and FChol, with increased MUFAs/PUFAs ratio and PtdCho-related ratios (namely, PtdCho/precursors, PtdCho/PtdEtn, and PtdCho/SM).

This work is not without limitations, mainly regarding the small sample size (which, if increased, would lend more solid statistical robustness to results) and the need of demonstration of the putative explanatory hypotheses advanced, mainly regarding HIR tumors, for which no enzyme expression data has yet been gathered. Additionally, as MG is rich in adipocytes, compared with tumor epithelial parenchyma, the changes observed between MG and tumors are likely to be a simple reflection of these differences; still, comparison with MG was useful to establish the basal HD metabolome. Despite the above limitations, we suggest that monitoring particular lipid families and specific lipid ratios may become helpful, not only to follow tumor progression to acquired ET-resistant stages but also to monitor the effectiveness of eventual metabolically targeted therapies to overcome that type of resistance.

## Data Availability

Data will be found on the Metabolomics Workbench, https://www.metabolomicsworkbench.org/ (accessed 06-26-2023) (submission under review), with data track identification number 4113.

## Supporting Information Available

Figure S1: Scores scatter plots obtained for (a) PCA and (b) PLS-DA of spectra recorded for lipid extracts corresponding to MG+MPA samples compared to MG samples; Figure S2: PCA scores scatter plots for pairwise sequential and nonsequential group comparisons; Figure S3: Proliferation index of the three tumor types; Table S1: Peak assignments corresponding to the resonances observed in the ^1^H NMR spectra of lipophilic extracts of MG and tumor tissues obtained from the MPA-induced mouse model of breast cancer; Table S2: Average lipid characteristics and lipid ratios for selected lipid classes (PDF)

## Notes

All animal procedures were performed at the IByME Animal facility and approved by the local Institutional Animal Care and Use Committee (approval no. 030/2016, dated June 24, 2016), complying with regulatory standards of animal ethics.

## Abbreviations

25HC: 25-hydroxycholesterol
27HC: 27-hydroxycholesterol
Abcc3: ATP-binding cassette subfamily C member 3
ACADS: Acyl-CoA dehydrogenase short chain
ACOX1: Acyl-CoA oxidase 1
ACSL3: Acyl-CoA synthetase long-chain family member 3
ApoE: apolipoprotein E
BC: breast cancer
*CCND1*: *Cyclin D1* protein coding gene
CDCl_3_: deuterated chloroform
Cer: ceramide
Cho: choline
α-KG: alpha-ketoglutarate
CDP-Cho: cytidine diphosphocholine
CDP-Etn: cytidine diphosphate ethanolamine
cMYC: cellular MYC, often overexpressed in several cancers
Cyp39a1: 24-hydroxycholesterol 7-alpha-hydroxylase
DHAP: dihydroxyacetone phosphate
ERα: estrogen receptor alpha
ET: endocrine therapy
Etn: ethanolamine
FA: fatty acid
FASN: FA synthase
FChol: free cholesterol
FDR: false discovery rate
FGF2: fibroblast growth factor 2
GPC: glycerophosphocholine
GTP: guanosine-5′-triphosphate
GTPase: GTP hydrolase
HADH2: 3-hydroxyacyl-CoA dehydrogenase type-2
HD: hormone-dependent
HI: hormone-independent
HIR: hormone-independent and resistant
HMDB: human metabolome database
HMG-CoA: 3-hydroxy-3-methylglutaryl coenzyme A
HMGCS1: 3-hydroxy-3-methylglutaryl-coenzyme A synthase 1
HR: hormone receptor
LA: linoleic acid
LDLR: low-density lipoprotein receptor
LDLRAP1: low-density lipoprotein receptor adapter protein 1
LTED: long-term estrogen deprived
LXR: liver-S-receptor
MG: mammary gland
MPA: medroxyprogesterone acetate
MS: mass spectrometry
MUFAs: monounsaturated fatty acids
MYC: transcription factor encoded by the MYC gene
NMR: nuclear magnetic resonance
PCA: principal component analysis
PECR: peroxisomal trans-2-enoyl-CoA reductase
PEX3: peroxisomal biogenesis factor 3
Pls: plasmalogen
PLs: phospholipids
PLS-DA: partial least-squares discriminant analysis
PR: progesterone receptor
PtdCho: phosphatidylcholine
PtdEtn: phosphatidylethanolamine
PUFAs: polyunsaturated fatty acids
PXMP2: peroxisomal membrane protein 2
SMs: sphingomyelins
SREBP: sterol regulatory element binding protein
TCA cycle: tricarboxylic acid cycle
TGs: triacylglycerides
TMS: tetramethylsilane
TP53: tumor protein p53
UDP-GlcNAc: uridine diphosphate *N*-acetylglucosamine
UV: unit variance
VIP: variable importance to the projection.
