# Supplementary material for: Tumor Lipid Signatures Are Descriptive of Acquisition of Therapy Resistance in an Endocrine-Related Breast Cancer Mouse Model
Source: J Proteome Res. 2023 Jul 27;23(8):2815–29. doi: 10.1021/acs.jproteome.3c00382 (PMC11301694; doi:10.1021/acs.jproteome.3c00382)
Supplement: Supplementary file 1 — pr3c00382_si_001.pdf [file pr3c00382_si_001.pdf]

Supplementary Information

Tumor lipid signatures are descriptive of acquisition of therapy resistance in an endocrine-related breast cancer mouse model

Rita Araújo[1], Victoria Fabris[2], Caroline A. Lamb[2], Andrés Elía[2], Claudia Lanari[2], Luisa A. Helguero[3,†,], Ana M. Gil[1,†,*]*

[1]Department of Chemistry and CICECO - Aveiro Institute of Materials (CICECO/UA), University of Aveiro, Campus Universitario de Santiago, 3810-193 Aveiro, Portugal

[2]IByME – Instituto de Biología y Medicina Experimental, Vuelta de Obligado 2490, C1428 ADN, Buenos Aires, Argentina

[3]iBIMED - Institute of Biomedicine, Department of Medical Sciences, Universidade de Aveiro, Agra do Crasto, 3810-193 Aveiro, Portugal

Figure S1. PCA scatter plots for pairwise sequential and non-sequential group comparisons.

Figure S2. Proliferation index of the three tumor types. The resonances observed in the ^{1}H NMR spectra of lipophilic extracts of MG and tumor tissues obtained from the MPA-induced mouse model of breast cancer.

Table S2. Average lipid characteristics and lipid ratios for selected lipid classes.

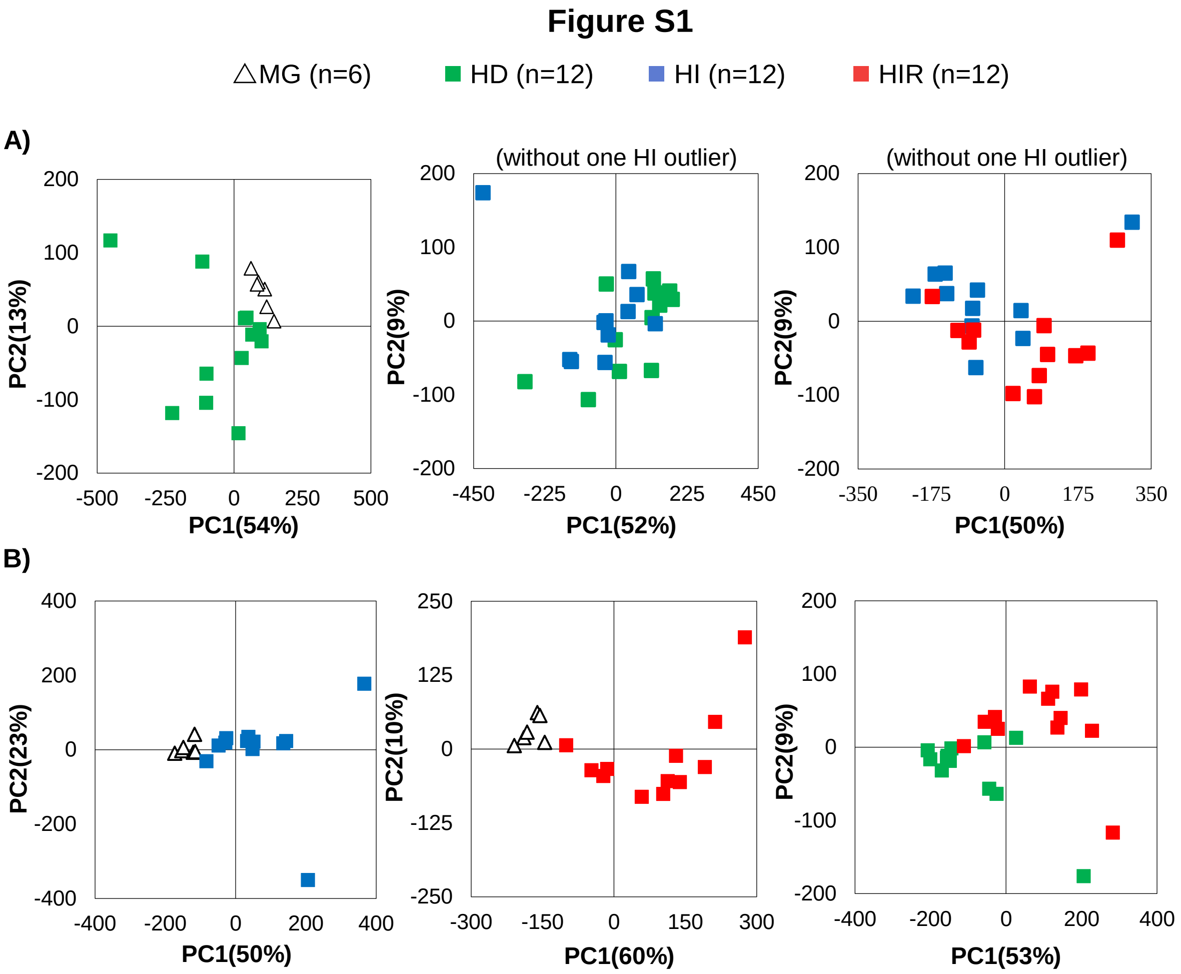

Figure S1. PCA scatter plots for pairwise sequential and non-sequential group comparisons. (A) PCA for sequential group comparisons: HD tumors *vs* MG tissue (healthy animals), HI tumors *vs* HD tumors and HIR tumors *vs* HI tumors (one HI tumor samples was removed from each model, as it was considered an outlier; however the same sample was maintained in PLS-DA and in all subsequent analysis), (B) PCA of non-sequential group comparisons: HI tumors *vs* MG tissue, HIR tumors *vs* MG tissue and HIR tumors *vs* HD tumors.

Figure S2

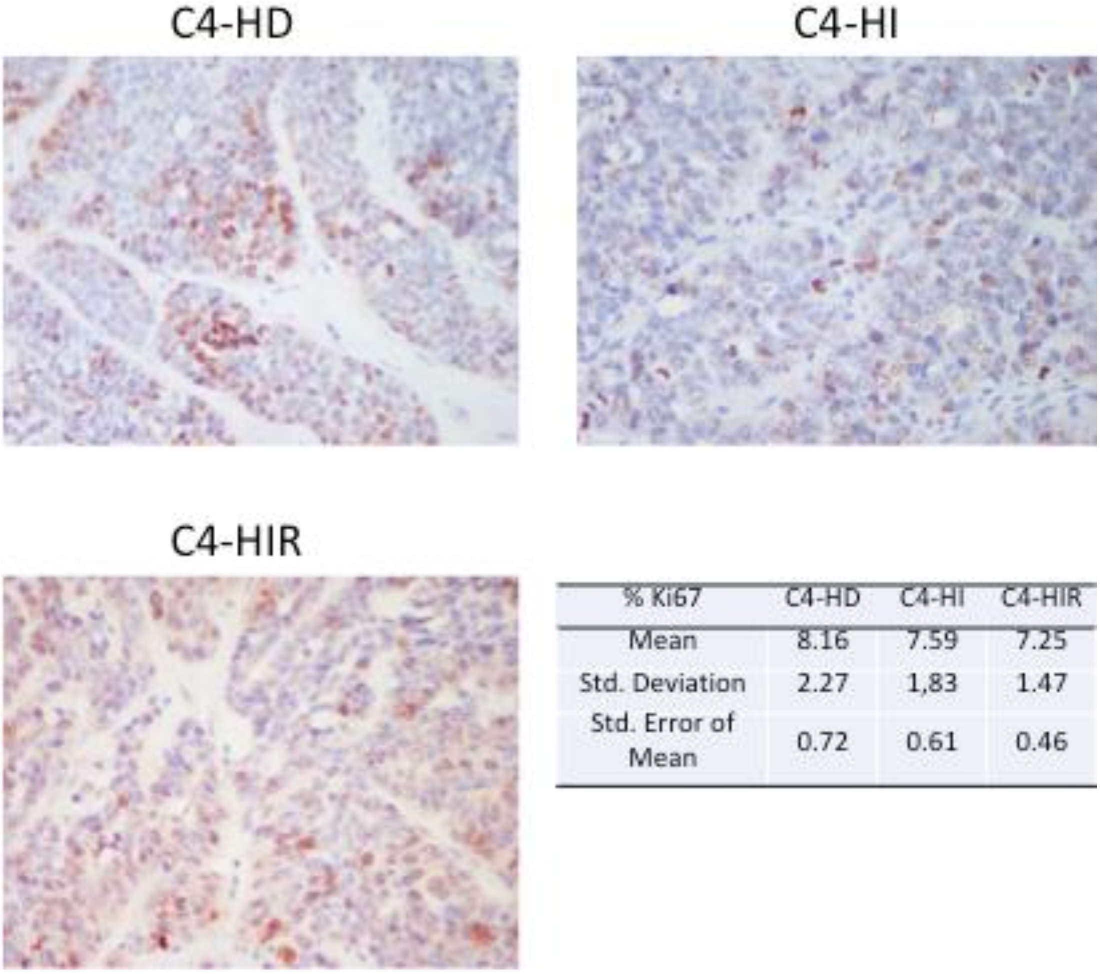

% Ki67	C4-HD	C4-HI	C4-HIR
Mean	8.16	7.59	7.25
Std. Deviation	2.27	1,83	1.47
Std. Error of Mean	0.72	0.61	0.46

Figure S2. Proliferation index of the three tumors. The panels show representative immunohistochemistry using the proliferation marker Ki67. The tumors were excised following 16 days-post implantations, immediately fixed in 10% formalin and processed for immunohistochemistry as described here [7].

Table S1. Peak assignments corresponding to the resonances observed in the ^{1}H NMR spectra of lipophilic extracts of MG and tumor tissues obtained from the MPA-induced mouse model of breast cancer. The assignment of lipid compounds is listed by the increasing chemical shift value of the first element of the corresponding spin system.

	**Metabolites**	**δ ^{1}H in ppm (multiplicity, assignment)**	**General relative amounts (tumor *vs* MG samples)**
**Sterols**	Lathosterol	0.53 (s,18-CH_3)	Only in tumors
	Free cholesterol	0.67 (s,18-CH_3), 0.85 (d,26-CH_3), 0.87 (d,27-CH_3), 0.91 (d,21-CH_3), 1.00 (s,19-CH_3), 1.03-1.19 (m, 1-CH_3, 12-CH_3, 15-CH_3, 17-CH_3, 23-CH_3, 24-CH_3), 1.40-1.55 (m, 7-CH_3, 8-CH_3, 15-CH_3, 25-CH_3), 1.78-1.87 (m, 1-CH_3, 2-CH_3, 16-CH_3), 1.93-2.03 (br, 7-CH_3, 12-CH_3), 2.19-2.32 (br,4-CH_3), 3.48-3.56 (br,3-CH_3), 5.33-5.36 (br,6-CH_3)	MG << tumors
**FAs**	All FAs	0.88 (t,-CH_3), 1.25-1.30 (m,(-CH_2)$_n$), 1.56-1.65 (m,-C<u>H</u>$_2$-CH_2-COO)	MG > tumors
	MUFAs	2.01-2.06 (m,-CH_2-C<u>H</u>$_2$-CH=)	
	FAs in TGs	2.30 (t,-CH_2-COO-)	
	FFAs	2.31 (t, -CH_2-COO-)	
	LA (18:2, ω-6)	2.77 (t, =CH-C<u>H</u>$_2$-CH=)	
	PUFAs	2.79-2.86 (m, =CH-C<u>H</u>$_2$-CH=)	
	UFAs	5.29-5.41 (m,-<u>H</u>C=C<u>H</u>-)	
**GLPs and TGs**	PtdEtn	3.11-3.18 (br, N-CH_2 of ethanolamine), 4.03-4.09 (br, PO-CH_2 of ethanolamine)	MG << tumors
	PtdCho	3.32 (s,- $N^+(CH_3)_3$ of choline)	
	PtdCho(+SMs)	3.71-3.75 (br, N-CH_2 of choline)	
	All GPLs	3.89-4.02 (m, PO-(3-CH_2) of glycerol), 4.33-4.43 (m, 1-CH_2 of glycerol), 5.19-5.23 (m, 2-CH of glycerol)	
	TGs	4.13 (dd, 1-CH_2/3-CH_2 of glycerol), 4.29 (dd, 1-CH_2/3-CH_2 of glycerol), 5.24-5.28 (m, 2-CH of glycerol)	MG > tumors
**SMs**	SMs	3.31 (s, -$N^+(CH_3)_3$), 5.68 (m, -CH_2-C<u>H</u>=CH-CHOH-)	MG << tumors
	SMs (+PtdCho)	3.71-3.75 (br, N-CH_2 of choline)	
**Pls**	Pls	3.85 (m, 1-CH_2 of glycerol), 5.16 (m, 2-CH_2 of glycerol), 5.90 (d, O-<u>H</u>C=CH)	MG << tumors

Abbreviations: FAs, fatty acids; MUFAs, monounsaturated FAs; Pls, plasmalogens, PtdCho, phosphatidylcholine; PtdEtn, Phosphatidylethanolamine; PUFAs, polyunsaturated FAs; SMs, sphingomyelins; TGs, triacylglyceride; UFAs, unsaturated FAs. Multiplicity: s, singlet; d, doublet; dd, doublet of doublets; ddd, doublet of doublets of doublets; t, triplet; q, quartet; m, multiplet; br, broad signal.

Table S2. Average lipid characteristics and ratios for selected lipid classes. All ratios were obtained from the average normalized peak areas.

Sample groups	Av. unsat. degree	Av. polyunsat. degree	MUFAs/ PUFAs	Av. chain length of FAs	PtdCho/ Cho	PtdCho /GPC	PtdEtn/ Etn	PtdCho/ PtdEtn	PtdCho/ SM	PtdCho/ FChol	SM/ FChol
Average ± standard deviation for each sample group											
MG	0.89±0.04	0.20±0.01	2.37±0.22	18.47±0.78	0.13±0.07	0.025±0.013	0.03±0.02	1.86±0.22	1.34±0.11	1.30±0.23	0.96±0.13
HD	0.76±0.10	0.15±0.03	2.86±0.58	15.52±1.75	0.74±0.75	0.048±0.029	0.12±0.09	1.74±0.45	0.98±0.26	0.98±0.25	1.01±0.10
HI	0.77±0.07	0.14±0.02	2.90±0.33	15.13±1.33	1.30±0.63	0.10±0.054	0.24±0.10	1.61±0.42	0.87±0.20	1.06±0.26	1.23±0.23
HIR	0.79±0.06	0.12±0.03	3.53±0.37	14.33±1.19	1.64±0.57	0.32±0.10	0.43±0.14	2.06±0.08	0.99±0.08	1.25±0.11	1.27±0.16
Significant *p*-values for each pairwise comparison											
HD *vs* MG	1.29E-03	2.15E-04	6.90E-03	4.31E-04	2.45E-02	-	2.05E-03	-	4.31E-04	1.82E-02	-
HI *vs* HD	-	-	-	-	1.45E-02	3.64E-03	3.64E-03	-	3.87E-02	-	1.2E-02
HIR *vs* HI	-	-	4.96E-04	-	7.80E-02	1.41E-05	2.32E-03	5.18E-06	4.49E-02	2.05E-02	-
HI *vs* MG	7.54E-04	1.08E-04	1.08E-04	2.16E-04	4.31E-04	1.29E-03	1.08E-04	-	1.08E-04	-	2.45E-02
HIR *vs* MG	2.05E-03	1.08E-04	1.08E-04	2.16E-04	1.08E-04	1.08E-04	1.08E-04	-	1.08E-04	-	2.05E-03
HIR *vs* HD	-	1.00E-02	4.96E-04	2.42E-02	2.32E-03	7.40E-07	2.19E-05	4.96E-05	-	1.03E-04	4.96E-04

Abbreviations: Cho, choline; Etn, ethanolamine; FAs, fatty acids; FChol, free cholesterol; GPC, glycerophosphocholine; MUFAs, monounsaturated FAs; PtdCho, phosphatidylcholine; PtdEtn, phosphatidylethanolamine; PUFAs, polyunsaturated fatty acids.
